# Revisiting the OGIPRO Trial: Dynamic Electronic Patient-Reported Outcomes Compared with EQ-5D-5L in HER2-Positive Breast Cancer

**DOI:** 10.3390/cancers18040614

**Published:** 2026-02-13

**Authors:** Anatol Aicher, Marcus Vetter, David Blum, Andreas Trojan

**Affiliations:** 1 Medical Faculty, University of Zurich, 8006 Zurich, Switzerland; anatol.aicher@uzh.ch (A.A.); david.blum@usz.ch (D.B.); 2 Medical Faculty, University Basel, 4001 Basel, Switzerland; marcus.vetter@ksbl.ch; 3 Cantonal Hospital Baselland, 4410 Liestal, Switzerland; 4 Department of Radiation Oncology, University Hospital Zurich, 8091 Zurich, Switzerland; 5 Brustzentrum Zürichsee, 8810 Horgen, Switzerland; 6 Mobile Health AG, 8008 Zurich, Switzerland

**Keywords:** ePRO, EQ-5D-5L, HER2-positive breast cancer, quality of life, QoL, digital monitoring, mixed-effects modeling, AI-ready, continuous, precision, real world, predictive, monitoring

## Abstract

Introduction: Patient-reported outcomes (PROs) are increasingly valued in oncology for capturing treatment tolerability and quality of life, and they are emerging as important data sources for precision-medicine and AI-driven clinical workflows. While the EQ-5D-5L questionnaire remains a widely used standardized instrument, dynamic electronic PROs (ePROs) collected via mobile applications generate richer, higher-frequency longitudinal data. Their alignment with established PRO measures, however, is not well-understood, limiting their integration into routine care and downstream analytic applications. In the prospective OGIPRO trial (KEK-ZH 2021-D0051), patients with HER2-positive breast cancer reported well-being and symptoms via the Medidux ePRO platform alongside weekly EQ-5D-5L assessments. In this retrospective analysis, we used linear mixed-effects modeling to examine associations between: (i) dynamic ePRO well-being and the EQ-5D-5L visual analogue scale (VAS); (ii) dynamic ePRO symptom grades and EQ-5D-5L domain sums; (iii) ePRO symptom grades and EQ-5D-5L disutility using the EQ-5D-5L value set for Germany. Materials and Methods: The analytic dataset comprised 13,699 dynamic ePRO data points (3376 well-being ratings and 10,323 symptom grades across 91 symptom types) from 53 patients, forming high-frequency longitudinal patient trajectories. Of these, 252 and 226 time-aligned observations, respectively, were used for direct comparison with EQ-5D-5L VAS and domain scores. Results: Dynamic ePRO well-being showed strong agreement with EQ-5D-5L VAS (β = 1.061, 95% CI: 1.015–1.107), with low between-patient variability. In contrast, the agreement between aggregated ePRO symptom grades and EQ-5D-5L domain sums was weaker (β = 0.404, 95% CI: 0.307–0.501) and more heterogeneous across patients. The same applied to the agreement between ePRO symptom grades and EQ-5D-5L disutility (β = 0.213; 95% CI: 0.151–0.275). Discussion: Dynamic ePRO well-being aligns closely with EQ-5D-5L VAS scores, supporting its use as a pragmatic substitute in clinical and research settings. Aggregated symptom grades, however, showed limited concordance with EQ-5D-5L domains, indicating the need for more granular analyses on larger datasets. Conclusions: Overall, dynamic ePRO systems provide validated, high-resolution longitudinal patient data and represent a scalable foundation for patient monitoring and data-driven decision support in oncology, including future AI-based precision-medicine applications.

## Simple Summary

Cancer treatments can affect patients’ daily lives in ways that are not always fully captured during clinic visits. To better understand how patients feel during therapy, doctors often use questionnaires, but these are usually completed only at fixed time points. New digital tools now allow patients to report their well-being and symptoms continuously using their smartphones, creating detailed, real-time pictures of how each patient is doing. In this study of people with a specific type of breast cancer, we compared these continuous digital patient reports with a widely used standard quality-of-life questionnaire. We found that patients’ self-reported overall well-being collected digitally closely matched the standard questionnaire results, showing that smartphone-based reporting can reliably reflect how patients feel. However, when many different symptoms were combined into a single summary score, the agreement was weaker, suggesting that symptoms need to be analyzed in a more detailed way. These findings show that digital patient reporting can provide reliable, high-resolution information about patients’ health. This type of data can improve how patients are monitored in everyday cancer care and support the future use of data-driven and AI-based tools to deliver more personalized, patient-centered treatment.

## 1. Introduction

Patient-reported outcomes (PROs) have become increasingly important in oncology, providing insights into treatment tolerability and patient well-being that extend beyond traditional clinical endpoints. In parallel, precision oncology and artificial intelligence (AI) approaches increasingly rely on longitudinal, patient-centered data streams to support individualized monitoring and timely clinical decision-making. However, for PRO-derived digital signals to be used confidently in such workflows, they must be anchored to established, clinically interpretable instruments.

The EQ-5D-5L, introduced by the EuroQol Group in 2011, is one of the most widely used standardized instruments for assessing health-related quality of life [1]. It evaluates five core dimensions—mobility, self-care, usual activities, pain/discomfort, and anxiety/depression—each on a five-level scale and includes a visual analogue scale (VAS) of overall health.

Electronic PROs (ePROs), particularly mobile-application-based systems, enable the remote, asynchronous, and structured collection of PROs. Dynamic ePRO describes a class of ePROs, where patients provide data on their own initiative, on their own time, thereby generating higher-frequency longitudinal trajectories than scheduled questionnaires. Prior studies have shown that dynamic ePRO monitoring facilitates the early detection of adverse events, supports timely clinical interventions, enhances patient–physician communication, and shows high adherence [2,3,4,5,6]. ePRO monitoring is feasible even in patients with advanced cancer [7]; digital interventions are increasingly accepted [7] and show the ability of improving symptom management, quality of life and survival [2,8].

Although dynamic ePROs offer advantages in data richness and timeliness, their relationship to established PRO instruments such as the EQ-5D-5L remains insufficiently characterized. Establishing congruence between continuous, patient-initiated ePRO signals and standardized measures is an important prerequisite for integrating such data streams into routine care and for enabling downstream precision-medicine applications, including individualized monitoring and predictive or decision-support models.

We therefore examined the alignment of dynamic ePRO reporting with EQ-5D-5L assessments in patients with HER2-positive breast cancer enrolled in the OGIPRO clinical trial (KEK-ZH 2021-D0051) [9]. Medidux, the dynamic ePRO tool used in the OGIPRO study, allows structured, CTCAE-aligned symptom [10] reporting across more than 110 categories. Using linear mixed-effects modeling, we investigated associations between (i) ePRO well-being and EQ-5D-5L VAS scores; (ii) ePRO symptom grades and EQ-5D-5L domain sums; (iii) ePRO symptom grades and EQ-5D-5L disutility using the EQ-5D-5L value set for Germany [11].

## 2. Materials and Methods

### 2.1. Study Design and Population

This analysis was conducted within the OGIPRO trial (KEK-ZH 2021-D0051), a non-interventional, multicenter, prospective observational study in Switzerland. Eligible participants were adults with HER2-positive breast cancer (early-stage, locally advanced, or metastatic) receiving systemic therapy according to national guidelines, see Table 1. Written informed consent was obtained from all patients. This study was approved by the Cantonal Ethics Committee Zurich and conducted in accordance with the Declaration of Helsinki.

### 2.2. Data Collection

Patients reported symptoms and overall well-being using the Medidux mobile application (version 3.2, mobile Health AG, Zurich, Switzerland), which allows collection of dynamic ePRO data. The app provides structured, CTCAE-based symptom reporting across more than 110 categories and includes a continuous well-being rating. Patients were encouraged to record entries as frequently as they deemed relevant.

In parallel, standardized EQ-5D-5L questionnaires were electronically administered approximately once weekly. Each EQ-5D-5L record included five domain-specific items—mobility, self-care, usual activities, pain/discomfort, and anxiety/depression—each graded on a five-level severity scale, and a visual analogue scale (VAS) reflecting overall health status.

**Figure 1:**
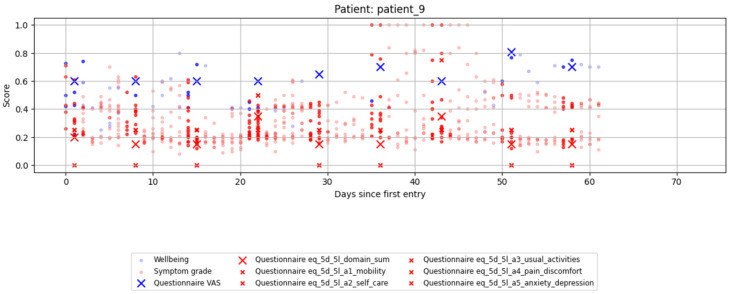
Example of data collection during the OGIPRO trial for a single patient. Crosses represent EQ-5D-5L questionnaire responses (blue: VAS, red: domain sum, small red: individual domains), normalized to a 0–1 scale. Dots represent dynamic ePRO entries (blue: well-being, red: symptom grades). Highlighted entries indicate ePRO data within ±1 day of an EQ-5D-5L response, which were used for analysis.

See Figure 1 for an example set of data.

### 2.3. Data Preprocessing

For comparative analysis, EQ-5D-5L entries were aligned with ePRO reports within a ±1-day window. When multiple ePRO entries fell within this window, averages were calculated separately for well-being and symptom grades. When no ePRO entries were available within this window, the EQ-5D-5L entries were not used in the analysis.

When comparing ePRO symptom grades to EQ-5D-5L domains, it should be noted that EQ-5D-5L domains are usually converted to a health score, called an index, using country-specific weights, called value-sets [1,12,13]. A value-set for Switzerland does not exist, with the German value-set being commonly used as a replacement. The index is calculated using:

(1) $$U=1-D=1-\sum d_{DOMAIN}\left(level\right)$$

Here, *U* is the resulting index value, *D* is the disutility, and $d_{DOM}\left(level\right)$ is the weight value for the EQ-5D-5L domain *DOMAIN* at a given level *level*. See Table 2 for the German value-set [11].

Since the ePRO symptom grades, the individual reported EQ-5D-5L levels, as well as the domain sum all increase with worsening health, but the index instead decreases with worsening health, we used only the disutility term, scaled by its hypothetical maximum value (for normalization), yielding *D′*:

(2) $$D=\sum d_{DOMAIN}\left(level\right)\;\;\;D^{\prime }=\frac{1}{1.661}\sum d_{DOMAIN}\left(level\right)\;$$

It is debatable whether the average of the ePRO symptom grades are more appropriately reflected by the EQ-5D-5L index or the simple domain sum. We decided to analyze both.

**Figure 2:**
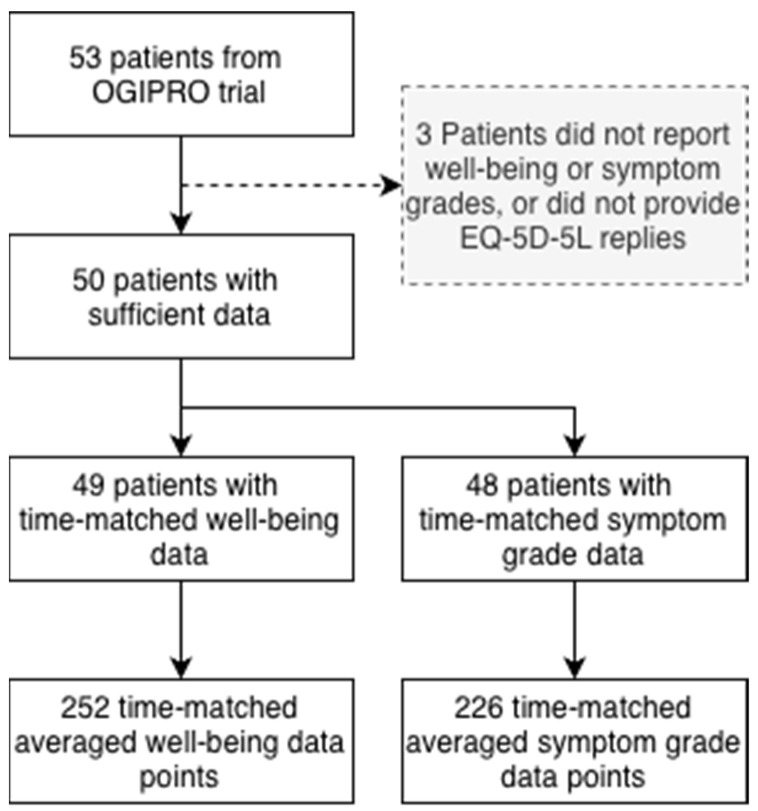
Out of 53 patients for whom data was available from the OGIPRO trial, 50 had sufficient data for analysis (meaning they had provided EQ-5D-5L replies and had entered either well-being or symptom grade data). Of the 50 patients, 49 had well-being entries within ±1 day of an EQ-5D-5L reply, and 48 patients had symptom grade entries within ±1 day of an EQ-5D-5L reply. After averaging well-being and symptom grades within the given time window, 252 and 226 time-matched data points were available.

The analytic dataset comprised a total of 3376 well-being and 10,323 symptom grade entries (across 91 different symptom types), for a total of 13,699 ePRO entries from 53 patients. Of these, 50 patients contributed data appropriate for analysis (i.e., both EQ-5D-5L and dynamic ePRO entries within matching time window), see Figure 2.

We averaged the symptom grades across all 91 symptom types into a single average symptom grade. While this reduced the richness of the dataset (see Section 4), performing a more detailed analysis would have required more advanced methods and likely a larger dataset.

After averaging well-being and symptom grades, a total of 252 matched observations from 49 patients were available for the analysis of well-being versus EQ-5D-5L VAS and 226 matched observations from 48 patients for symptom grade versus EQ-5D-5L domain sum/disutility.

All dynamic ePRO and EQ-5D-5L values were normalized to a 0–1 scale. Higher dynamic ePRO symptom grades correspond to greater symptom burden, while higher well-being values represent better well-being: This corresponds to the directionality of EQ-5D-5L, where higher domain grades correspond to greater disutility, but a higher VAS corresponds to better subjective health.

### 2.4. Statistical Analysis

**Figure 3:**
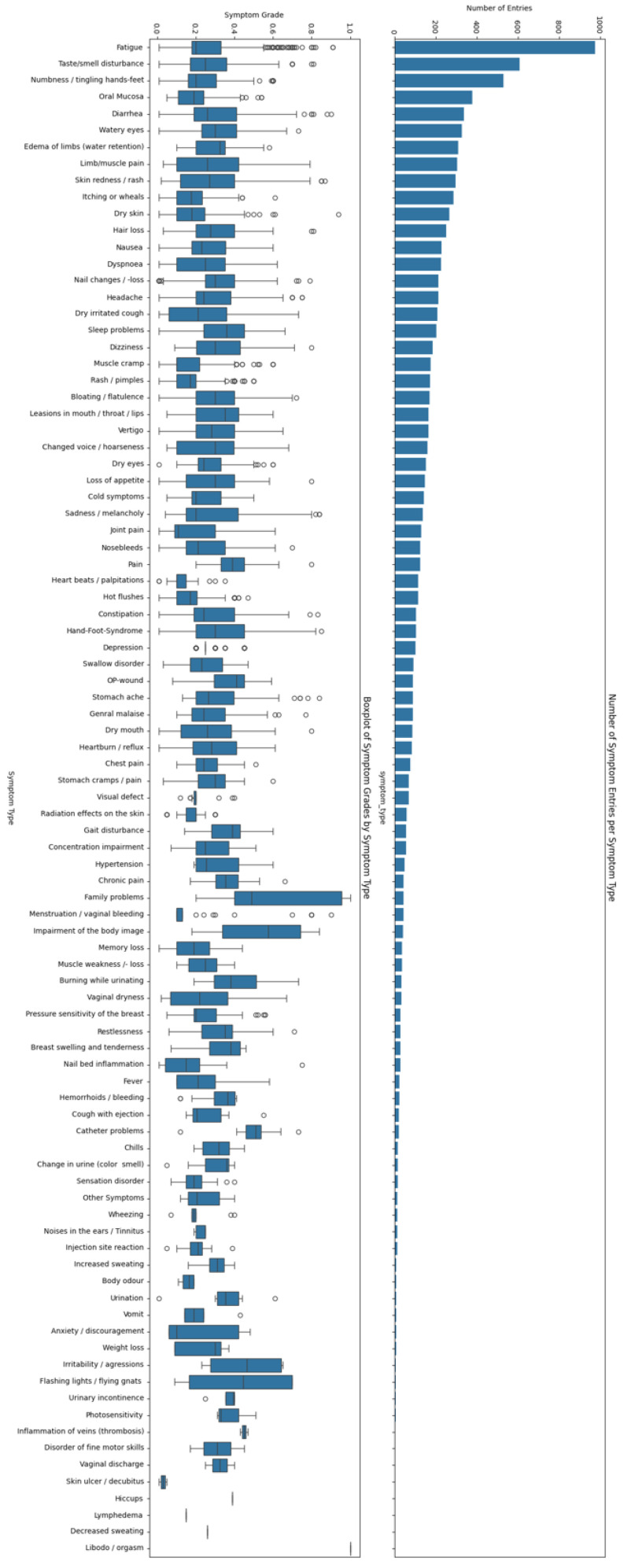
Histogram and box plot of the symptom entries (white circles) provided by patients. The x-axis lists all symptom types (as available in the Medidux app 3.2.10) that patients provided entries for. The histogram reports the total number of entries per type. The bar plot reports the average, interquartile range, and 1.5 interquartile range whiskers. For correlating EQ-5D-5L domain sum with symptom grade, all symptom entries within ±1 day of an EQ-5D-5L entry were averaged.

Associations between dynamic ePRO measures and EQ-5D-5L outcomes were assessed using linear mixed-effects models to quantify both population-level associations and patient-specific deviations in longitudinal PRO trajectories, see Figure 3. We chose a relatively simple over a complex model for interpretability and reproducibility,] and to avoid overfitting and generating a false positive result, in accordance with recent guidance [14].

For the first model, the EQ-5D-5L VAS score was modeled as a function of dynamic ePRO well-being:

(3) $$y_{ij}=\beta \cdot x_{ij}+u_{i}\cdot x_{ij}+\varepsilon_{ij}$$

where *y_ij_* and *x_ij_* are the VAS and well-being entries for patient *i* at observation *j*, β is the fixed (population-level) slope, *u_i_* is the patient-specific deviation (random effect) from the population slope, and *ε_ij_* is the residual error term.

For the second model, the EQ-5D-5L domain sum was modeled as a function of dynamic ePRO symptom grade, using the same model, with *y_ij_* and *x_ij_* being the EQ-5D-5L domain sum and symptom grades for patient i at observation j.

For the third model, we used the EQ-5D-5L disutility (see Equation (2)) instead of the EQ-5D-5L domain sum.

Estimated coefficients *β* and *u*, standard errors, and 95% confidence intervals were derived using Restricted Maximum Likelihood (REML) analysis. All analyses were conducted using Python (version 3.13.1, Python Software Foundation, Wilmington, DE, USA) with numpy (version 2.4.2) [15], pandas (version 3.0.0) [16], and the statsmodels package (v 0.14.5) [17]. Plots were created using matplotlib (version 3.10.8) [18] and seaborn (version 0.13.2) [19].

## 3. Results

### 3.1. Well-Being vs. EQ-5D-5L VAS Score

A total of 252 observations were analyzed (averages of well-being entries within ±1 day of EQ-5D-5L replies versus EQ-5D-5L VAS score), with minimum, mean, and maximum group sizes (number of matched pairs per patient) of 1, 5.1, and 10, respectively.

**Figure 4:**
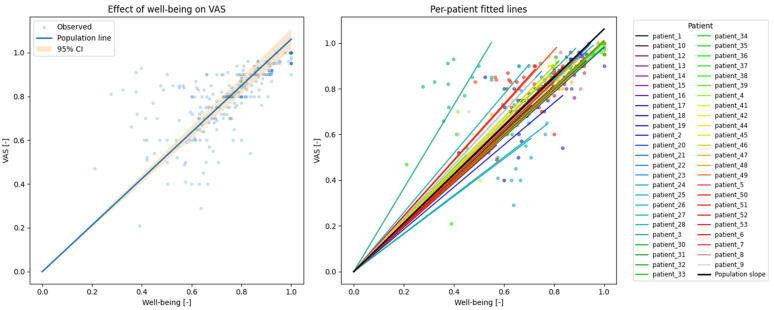
Agreement between normalized dynamic ePRO well-being and EQ-5D-5L VAS. (**Left**) Normalized dynamic ePRO well-being vs. normalized EQ-5D-5L VAS values. The line shows the slope of the mixed-effects regression (β = 1.061) with the confidence intervals colored orange (β ranges from 1.015 to 1.107). The graph shows relatively good agreement between the dynamic ePRO well-being and VAS. (**Right**) Per-patient slopes, showing good agreement/low variability between patients (u = 0.023 ± 0.070).

Mixed-effects model regression for VAS vs. dynamic ePRO well-being yielded a coefficient β = 1.061 ± 0.024; 95% CI: 1.015–1.107. The variability between groups (i.e., patient-specific deviation from the population slope u) was 0.023 ± 0.070. For visual representation, see Figure 4.

### 3.2. Symptom Grade vs. EQ-5D-5L Domain Sum

A total of 226 observations were analyzed (averages of symptom grade entries within ±1 day around EQ-5D-5L replies versus EQ-5D-5L domain sum), with minimum, mean, and maximum group sizes (number of matched pairs per patient) of 1, 4.7, and 10, respectively.

**Figure 5:**
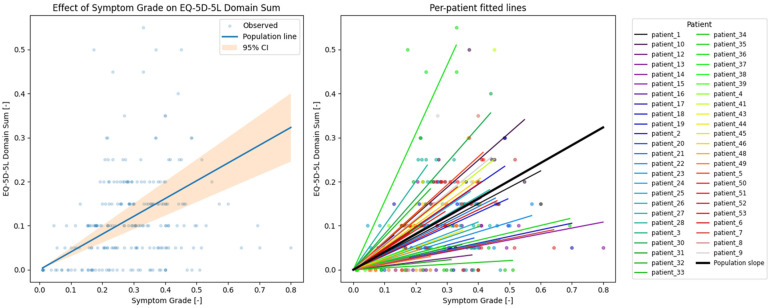
Relationship between normalized dynamic ePRO symptom grades and EQ-5D-5L domain sums. (**Left**) Normalized dynamic ePRO symptom grade vs. normalized EQ-5D-5L domain sum values. The line shows the slope of the mixed-effect regression (β = 0.404) with the confidence intervals colored orange (β from 0.307 to 0.501). The graph shows low agreement between dynamic ePRO symptom grades and EQ-5D-5L domain sum. (**Right**) Per-patient slopes, showing high variability between patients (u = 0.093 ± 0.394).

Mixed-effects model regression for EQ-5D-5L domain sum vs. dynamic ePRO symptom grade yielded a coefficient β = 0.404 ± 0.049; 95% CI: 0.307–0.501. The variability between groups (i.e., patients-specific deviation from the population slope u) was 0.093 ± 0.394. For visual representation, see Figure 5.

### 3.3. Symptom Grade vs. EQ-5D-5L Disutility

A total of 226 observations were analyzed (averages of symptom grade entries within ±1 day around EQ-5D-5L replies versus EQ-5D-5L disutility), with minimum, mean, and maximum group sizes (number of matched pairs per patient) of 1, 4.7 and 10, respectively.

**Figure 6:**
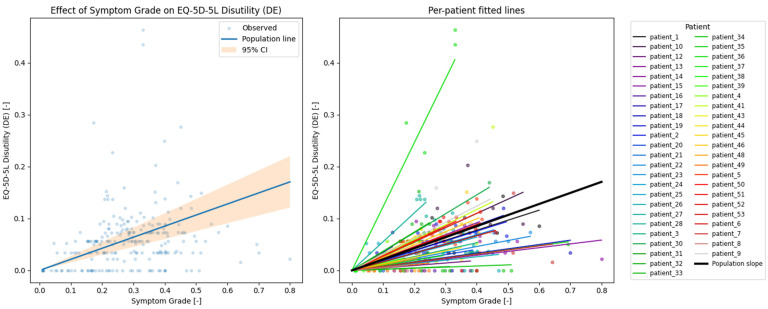
Relationship between normalized dynamic ePRO symptom grades and EQ-5D-5L disutility. (**Left**) Normalized dynamic ePRO symptom grade vs. normalized EQ-5D-5L disutility. The line shows the slope of the mixed-effect regression (β = 0.213) with the confidence intervals colored orange (β from 0.151 to 0.0.275). The graph shows low agreement between the dynamic ePRO symptom grades and EQ-5D-5L disutility. (**Right**) Per-patient slopes, showing the high variability between patients (u = 0.041 ± 0.322).

Mixed-effects model regression for EQ-5D-5L domain sum vs. dynamic ePRO symptom grade yielded a coefficient β = 0.213 ± 0.032; 95% CI: 0.151–0.275. The variability between groups (i.e., patient-specific deviation from the population slope u) was 0.041 ± 0.322. For visual representation, see Figure 6.

For a tabular view of the model results for all 3 models, see Table 3.

## 4. Discussion

This study provides the first real-world comparison of continuous ePRO monitoring with EQ-5D-5L questionnaires in patients with HER2-positive breast cancer receiving systemic therapy. By generating high-frequency, patient-initiated longitudinal data, the ePRO system captured a richer and denser dataset than scheduled questionnaires, underscoring its potential for supporting individualized patient monitoring and data-driven clinical workflows.

Our findings demonstrate that self-reported well-being determined via Medidux aligns closely with EQ-5D-5L VAS scores, with nearly one-to-one correspondence and minimal between-patient variability. This indicates that dynamic ePRO well-being represents a robust and clinically interpretable digital phenotype that can be reliably used as a substitute for EQ-5D-5L VAS collection in both clinical practice and research, providing a validated high-resolution input for continuous patient monitoring and downstream analytical applications.

By contrast, the relationship between the aggregated ePRO symptom grades and EQ-5D-5L domains was weaker and more variable. This held true regardless of using the EQ-5D-5L domain sum or country-corrected index. This heterogeneity likely reflects the loss of information introduced by aggregation: Medidux captures detailed, multi-dimensional symptom profiles across many categories, whereas the EQ-5D-5L compresses patient experiences into five broad domains, and our analysis further reduced both to single summary measures. Collapsing such complex, high-dimensional patient-reported data into global averages may obscure clinically relevant, patient-specific symptom patterns. It is plausible that specific symptoms correlate more strongly with some EQ-5D-5L domains than with others, e.g., the dynamic ePRO fatigue symptom probably more strongly correlates with the anxiety/depression or the mobility domain than with the pain/discomfort domain. Since 91 symptom types × 5 EQ-5D-5L domains yielded 455 potential correlations, we decided against analyzing them with mixed-effects models. This rich data lends itself to more advanced analytics, which should be explored in future research.

Another important limitation is the fact that we did not control for additional factors such as age, performance status, cancer stage, comorbidities, or other individual characteristics that may also contribute to the observed variability between patients and where there may be group differences. Given the small sample size, we consciously decided against it.

Lastly, the population analyzed consist entirely of women from Switzerland with HER2-positive breast cancer. This limits the generalizability of the results.

Future studies should examine symptom–domain relationships with finer granularity and in larger cohorts, enabling more detailed mapping between specific symptom clusters and quality-of-life domains. In this context, machine learning and other advanced AI methods may be particularly well-suited for modeling the high-dimensional, longitudinal structure of dynamic ePRO data, identifying clinically meaningful patterns, and capturing patient-specific trajectories that cannot be resolved through global summary scores alone. Such approaches may be required for fully leveraging continuous ePRO data streams in precision oncology, where individualized symptom dynamics can inform personalized monitoring strategies and provide data-driven clinical decision support. We believe that integrating dynamic ePROs with other digital biomarkers, wearable device data, and electronic health records is a promising area of future research.

## 5. Conclusions

Dynamic ePRO reporting via Medidux shows strong concordance with EQ-5D-5L well-being measures while providing higher temporal resolution and richer clinical context. These results support the use of dynamic ePRO well-being data as a pragmatic replacement for EQ-5D-5L VAS in both clinical practice and research, enabling continuous, patient-centered monitoring. For domain-level outcomes, further methodological refinement and larger datasets are required before dynamic ePRO symptom grades can be substituted for EQ-5D-5L domains.

Beyond questionnaire replacement, dynamic ePRO systems represent a scalable source of longitudinal, patient-generated data that is well-suited for precision oncology applications. By providing validated, high-frequency digital phenotypes, such systems can support advanced analytic approaches, including predictive modeling and AI-driven decision support, with the potential to improve individualized patient management and clinical outcomes.

## Figures and Tables

**Table 1:** Patient characteristics in the OGIPRO dataset, adapted from [9]. ECOG PS stands for Eastern Cooperative Oncology Group Performance Status. ICD refers to the International Classification of Disease.

Sex, *n* (%)	
F	53 (100)
Age (years)	
Mean (SD)	51.3 (10)
Median (range)	51 (31–78)
Primary ICD-10 Diagnosis, *n* (%)	
C50: malignant neoplasm (cancer) of the breast	53 (100)
Tumor stage T, *n* (%)	
T1	8 (21.05)
T2	19 (50.0)
T3	8 (21.05)
T4	3 (7.89)
Treatment setting, *n* (%)	
Neoadjuvant	17 (44.74)
Adjuvant	18 (47.37)
Palliative	3 (7.89)
Treatment, *n* (%)	
Trastuzumab	1 (2.63)
Trastuzumab + pertuzumab	21 (55.26)
Trastuzumab + pertuzumab + paclitaxel	13 (34.21)
Ado-trastuzumab emtansine	3 (7.89)
ECOG PS, *n* (%)	
0	13 (34.21)
1	15 (39.47)
2	6 (15.79)
3	2 (5.26)
4	2 (5.26)

**Table 2:** Value-set for EQ-5D-5L for Germany, based on [11]. These are used with Equation (1) to calculate the EQ-5D-5L index.

$d_{DOMAIN}\left(Level\right)$		Domain Level				
		1	2	3	4	5
EQ-5D-5L domain	Mobility	0.000	0.026	0.042	0.139	0.224
	Self-care	0.000	0.050	0.056	0.169	0.260
	Usual activities	0.000	0.036	0.049	0.129	0.209
	Pain/discomfort	0.000	0.057	0.109	0.404	0.612
	Anxiety/depression	0.000	0.030	0.082	0.244	0.356

**Table 3:** Results of mixed-effects modeling of 3 models.

Model	Domain Sum	Disutility Index	VAS
Response	EQ-5D-5L domain sum (domsum)	EQ-5D-5L disutility index (index)	EQ-5D-5L VAS (vas)
Predictor	ePRO symptom grade (grade)	ePRO symptom grade (grade)	ePRO well-being (well_being)
Formula	domsum~ 0 + grade	index~ 0 + grade	vas~ 0 + well_being
Fixed Effect	0.4040393	0.21313014	1.0609299
Fixed Effect SD	0.04941207	0.03154565	0.02366531
Fixed Effect *p*-Value	<10 × 10^−15^	1.42 × 10^−11^	<10 × 10^−15^
Random Effect	0.09255993	0.04053366	0.02292337
Random Effect SD	0.30423664	0.20132972	0.15140464
Number of Observations	226	226	252
Number of Groups (Patients)	48	48	49

## Data Availability

The data used in this study is available upon request and in accordance with the data sharing statement from the OGIPRO trial ethics approval.

## Abbreviations

The following abbreviations are used in this manuscript:

AI	Artificial Intelligence
CI	Confidence Interval
CTCAE	Common Terminology Criteria for Adverse Events
ePRO	Electronic Patient-Reported Outcome
EQ-5D-5L	EuroQol 5-Dimension 5-Level Questionnaire
HER2	Human Epidermal Growth Factor Receptor 2
ICD	International Classification of Disease
PRO	Patient-Reported Outcome
QoL	Quality of Life
REML	Restricted Maximum Likelihood
SD	Standard Deviation
VAS	Visual Analogue Scale
ECOG PS	Eastern Cooperative Oncology Group Performance Status.
